# Tick-Borne Encephalitis Vaccination Protects from Alimentary TBE Infection: Results from an Alimentary Outbreak

**DOI:** 10.3390/microorganisms9050889

**Published:** 2021-04-21

**Authors:** Lidia Chitimia-Dobler, Alexander Lindau, Rainer Oehme, Malena Bestehorn-Willmann, Markus Antwerpen, Marco Drehmann, Thomas Hierl, Ute Mackenstedt, Gerhard Dobler

**Affiliations:** 1 Bundeswehr Institute of Microbiology, 80937 Munich, Germany; lydiachitimia@gmail.com (L.C.-D.); MarkusAntwerpen@bundeswehr.org (M.A.); 2 Department of Parasitology, University of Hohenheim, 70599 Stuttgart, Germany; alexander.lindau@uni-hohenheim.de (A.L.); Malena1Bestehorn-Willmann@bundeswehr.org (M.B.-W.); marco.drehmann@uni-hohenheim.de (M.D.); mackenstedt@uni-hohenheim.de (U.M.); 3 State Health Office Baden-Württemberg, 70191 Stuttgart, Germany; rainer.oehme@rps.bwl.de; 4 District Health Office Zollernalbkreis, 72379 Hechingen, Germany; Thomas.Hierl@Zollernalbkreis.de

**Keywords:** tick-borne encephalitis virus, goat milk, alimentary infection

## Abstract

In May 2017, a hospitalized index case of tick-borne encephalitis (TBE) was confirmed by Serology. The case was linked to alimentary infection by raw milk from a goat farm in the region of Tübingen, Baden-Württemberg, Germany, where no previous TBE cases in the area had been reported before. The TBE focus was confirmed by isolation of the TBE virus from ticks and Serological confirmation of past infection in one of the five flock goats. Additional investigations by the local public health office identified 27 consumers of goat milk at the putative period of exposure. For 20/27 exposed persons, anamnestic information was gained by the local public health office. Twelve/fourteen exposed and non-vaccinated people developed clinical illness and were confirmed as TBE cases by Serology. Five/six vaccinated and exposed people did not develop the disease. The one exposed and vaccinated person had their last TBE vaccination booster more than 15 years ago, and therefore a booster was more than 10 years overdue. None of the regularly vaccinated and exposed persons developed clinical overt TBE infection. We report the first known TBE outbreak, during which, protection by TBE vaccination against alimentary TBE infection was demonstrated.

## 1. Introduction

Tick-borne encephalitis (TBE) is the most important tick-borne viral disease in Europe and Asia. Up to 10,000 human cases are reported annually. However, it is assumed that many mild and subclinical infections remain undiagnosed, and both the infection and the disease are supposedly highly under-reported [1]. TBE is caused by tick-borne encephalitis virus (TBEV), a member of the genus *Flavivirus* in the family *Flaviviridae* [2]. There are three accepted genetic subtypes and at least two other subtypes, the Baikalian subtype, and the Himalayan subtype, which were recently genetically proposed [3,4]. To date, only the European subtype has been detected circulating in Central European countries (Austria, Czech Republic, Germany, Slovak Republic, Switzerland) in ticks, rodents, and patients since the 1950s. TBEV circulates between ticks and natural hosts (small mammals) in strictly geographically limited natural foci [5].

The main route of TBEV infection is through a tick bite. However, alimentary transmission via non-treated dairy products is also a known way of TBEV transmission [6]. Milk-borne transmission was a common way of transmission in 1950s Czechoslovakia, and the related disease was named ‘biphasic milk fever’ [7]. Since then, milk-borne TBE outbreaks have been repeatedly reported in a number of central and eastern European countries [8,9,10,11,12,13,14,15]. Therefore it is recommended to pasteurize or boil milk before consumption [16]. Due to the increasing problem of alimentary infection in various countries, a vaccine candidate for small ruminants has been proposed [17]. TBE in animals is not yet well understood, and the knowledge of the pathogenesis of TBE in domestic animals is limited [18]. However, symptomatic infection has been reported in dogs, monkeys, and horses. Seroconversion without specific neurological symptoms of TBE has been described in ruminants such as cattle, goats, and sheep [19,20,21]. Information about TBE in domestic animals has recently been summarized [22]. Ruminants, especially goats, are known to shed TBEV in their milk, and the alimentary route of transmission is a well-accepted way of transmission, causing repeated outbreaks in humans [23]. Alimentary TBE outbreaks were rarely observed in countries with a highly industrialized milk industry. However, in recent years, TBE outbreaks caused by goat cheese have occurred in a mountainous region in Austria in 2008 [9] and in Germany in 2016 [11].

We investigated a cluster of 14 human TBE cases that occurred in May 2017 in southern Germany in consumers of raw goat milk from a goat kept in a flock of 5five animals. After the clinical diagnosis of meningoencephalitis and Serological confirmation of TBE in a hospitalized patient with potential milk-borne exposure, the local public health office was informed. A retrospective outbreak investigation was initiated in order to identify the source of infection and the extent of the outbreak. The aim of the investigation was to identify potential additional human cases and the extent of the outbreak, to study the seroprevalence of anti-TBEV antibodies in goat sera of the school farm, to identify the natural focus of the TBEV on the goat farm, and to isolate the TBEV from ticks and genetically characterize the virus through the natural focus.

## 2. Material and Methods

### 2.1. Human Infections

An active search to find consumers of goat milk by asking for milk consumption on a respective day was conducted by the local public health office. Serological tests for TBEV IgM and IgG antibodies were used to identify human infection. The results were confirmed by the German consulting laboratory in Munich. A human case was defined as exhibiting positive results of IgM and IgG against TBEV in association with the consumption of goat milk and having no vaccination against TBE. To exclude false-positive results caused by cross-reactivity to other flaviviruses, all sera were tested for IgM and IgG antibodies against different flaviviruses (TBEV, West Nile virus, Dengue virus, yellow fever virus, Japanese encephalitis virus) by indirect immunofluorescence assay (IFA) (Flavivirus Microchip 1, Euroimmun, Lübeck, Germany) according to standard procedures.

### 2.2. Tick Sampling and Natural Focus Identification

The goat flock was corralled in a small area, and the animals were only able to reach small bushes at the fence of the coral. Ticks were collected by dragging or flagging white linen flags through the vegetation around the flock stable, in the whole area, and in the surrounding forest. Ticks were tested according to the location in which the sample was taken. Moreover, this was performed for a detailed retrospective identification of the natural focus using the presumed place of goat infection with the positive tick pools.

### 2.3. Goat Flock Serology

A total of five adult goats were kept, mainly for educational purposes. The goats’ serostatus was tested to identify the infected goats 30 days after the infection of the index case. Blood samples were taken from each animal and tested by IFA using the flavivirus mosaic I microchip (Euroimmun, Lübeck, Germany) and anti-goat FITC conjugate (Dako, Glostrup, Denmark). A serum titer of ≥10 counted as positive. For confirmation, a virus neutralization test was performed. The plaque reduction neutralization test (PRNT) was performed using strain Neudörfl. Fifty plaque-forming units were added to the serum dilution 1:10 to 1:320 and incubated for 60 min at 37 °C. The virus–serum mixture was added to confluent A549 cells and overlaid with methylcellulose 0.75%. Plaques were counted after five days of incubation at 37 °C. A serum dilution reducing plaques at 90% was taken as a plaque-reduction neutralizing titer at 90% (PRNT_90_).

### 2.4. Virus Isolation and Characterization

Ticks were pooled according to species, life stages (three to ten nymphs and two to five adult ticks per pool), and their sampling location. Ticks were homogenized using 1 mL Minimum Essential Medium (MEM, Invitrogen, Karlsruhe, Germany) containing 10× Antibiotic-Antimycotic solution (ABAM, Invitrogen, Karlsruhe, Germany) using the Fast Prep Savant FP120 TissueLyser (Bio101, Vista, CA., USA), with three rounds at speed 6.5 for 30 s each. Total nucleic acid was extracted using the MagNA Pure LC RNA/DNA Kit (Roche, Mannheim, Germany) in a MagNA Pure LC instrument (Roche, Mannheim, Germany) according to the manufacturer’s instructions. The total nucleic acid was extracted in 50 µL, and a 5 µL aliquot was tested for TBEV RNA using a real-time RT-PCR (RT-qPCR) [24].

A 100 μL aliquot of the supernatants of crushed RT-qPCR-positive tick pools was given to an 80% confluent cell culture of A549 cells (human lung carcinoma cells, German Collection of cell cultures, DSMZ, Braunschweig). Each positive supernatant was used undiluted and in a dilution of 1:5 and 1:10. After one hour of incubation at 37 °C, the supernatant was pipetted off, and the cells were washed three times with MEM containing 5× ABAM and 3% fetal calf serum (Invitrogen, Karlsruhe). For cultivation, 5 mL of MEM containing 5× ABAM and 3% of fetal calf serum were added. Cells were incubated for up to seven days at 37 °C and observed daily for the occurrence of a cytopathic effect (CPE). In the case of more than 50% CPE, the supernatant was taken and tested by RT-qPCR for TBEV, as described. In case of no CPE culture, the supernatant was taken after seven days of incubation and also tested for growth of TBEV by RT-qPCR. No subcultures were conducted. From the isolated TBEV strains, E genes were sequenced for confirmation (see below).

The E genes were amplified directly from positive tick nucleic acid extractions and PCR-positive cell culture supernatants using conventional PCR [25]. A 1687 bp fragment was amplified and then sequenced using the outer primers and an additional internal sequencing primer, as described previously [25]. The products were purified after gel electrophoresis and processed as described [26]. All sequence data were processed using the software Geneious 9.1.5. A de novo assembly was performed using the three chromatograms obtained from GATC (Eurofins Genomics, Ebersberg, Germany) for each positive sample. Nucleotides with an estimated error higher than one percent were trimmed. Subsequently, the sequences were cut to 1488 bp, the exact length of the envelope gene sequence.

The evolutionary history was inferred and visualized using the Maximum Likelihood method and Tamura-Nei model implemented in the software MEGA X. For this, a ClustalW alignment of the E gene of representative isolates was generated. As a test of phylogeny, a bootstrap analysis was performed with 1000 replicates [3], and a further phylogenetic tree was generated using the PhyML algorithm [27].

## 3. Results

### 3.1. Human Infection

**Figure 1:**
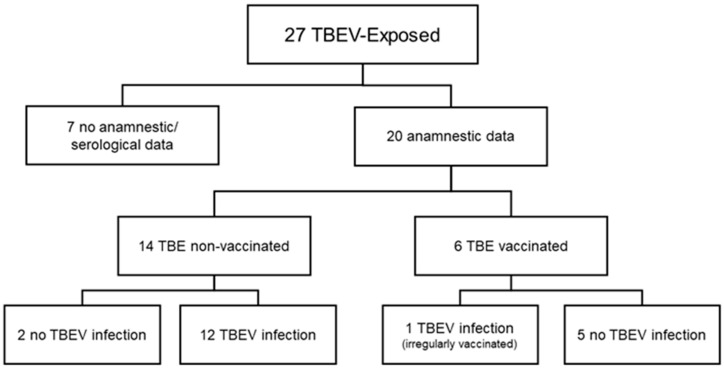
Number and proportion of exposed and infected persons.

A human case of TBE was diagnosed at the beginning of May 2017 and reported to the district public health office. This was the index case of the outbreak and the only patient that was hospitalized. The epidemiological investigation by the district public health office revealed the possibility of a goat milk outbreak of TBE. Therefore, a retrospective investigation to detect more possibly exposed patients during the period of exposition (1st to 7th of April 2017) was initiated by the district health office. As a result, a total of 27 people who drank goat milk on the respective days were identified (Figure 1). For 7/27 exposed persons, no data were available. For the remaining 20 exposed people, medical information was available for further analysis. Thirteen/twenty persons were infected, as shown by the detection of anti-TBEV IgM and a significant increase in anti-TBEV IgG. No specific IgM against any of the other tested flaviviruses was detected in the sera of the infected patients. Some low IgG cross-reaction against other flaviviruses was detected; the anti-TBEV IgG titer, however, in all patients was four-fold or higher compared to the other tested flaviviruses. All 13/20 infected persons reported unspecific febrile generalized flu-like symptoms, including headache, muscle ache, neck stiffness, gastrointestinal symptoms, prostration, and dizziness. All symptoms started between 14 and 20 days after the exposition to goat milk. In 7/20 exposed persons with no detected TBE infection, no clinical symptoms were reported, and no anti-TBEV IgM was detected in any of the sera.

The medical TBE vaccination history of the exposed persons revealed that 6/20 exposed people were vaccinated against TBE. Of these exposed and vaccinated patients, only one patient developed symptoms, and a TBEV infection was proven serologically. Fourteen/twenty exposed people were not vaccinated against TBE. Twelve/Fourteen exposed and non-vaccinated patients developed symptoms, and finally, a TBEV infection was serologically confirmed. In conclusion, 12/14 (86%) of the exposed and non-vaccinated persons developed a TBEV infection, while only in 1/6 (17%) of the vaccinated and exposed persons was a TBEV infection confirmed. All medical vaccine records of the exposed persons were checked by the public health office, and it was shown that the last TBE vaccination of the vaccinated and fallen-ill person was overdue by more than ten years.

### 3.2. Tick Sampling and Virus Characterization

After being informed about the TBE milk outbreak, a tick sampling activity was initiated in the whole affected area around the goat stable, and the meadows the goats were reported to use. On the 18th of May and the 1st of June 2017, ticks were sampled by flagging or dragging in the forests and meadows around the goat stable. On the 18th of May, 189 ticks (all *Ixodes ricinus*) were flagged. One nymph tick pool (1/189, minimal infection rate (MIR) 0.53%)) tested TBEV positive. On the 1st of June, 277 ticks could be collected. Two adult female pools were found to be TBEV positive (2/277, MIR 0.72%). The two positive tick pools were localized to a sampling place near a bushy areal, directly by the goat corral, but separated by a fence from the goat stable. All other areas around the areal, including the meadows, exhibited high numbers of ticks, but none of the sampled ticks there tested positive.

**Figure 2:**
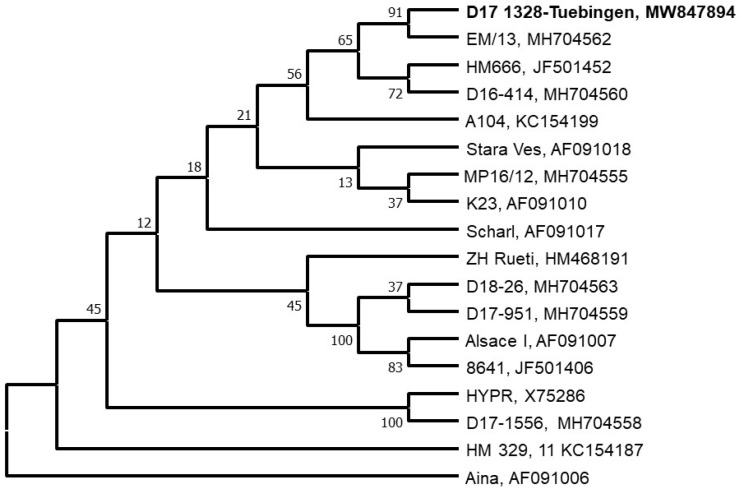
Maximum-likelihood-Analysis of E-Gen of the TBEV-EU isolate of the milk outbreak. The isolate of the outbreak (“Tübingen”) is shown in bold.

From the two female tick pools, TBEV could be successfully isolated in cell culture. The E gene sequences of the two TBEV strains could be completely sequenced directly from the positive female tick pools and the cell culture isolates. The sequences revealed a 100% identity between the tick and cell culture sequences. A phylogenetic analysis revealed a close phylogenetic relationship to recent TBEV strains in two neighboring areas (Emmendingen, Wutach) and a TBEV cluster in eastern Bavaria (Haselmühl) (Figure 2). More distantly, the strain was related to a TBEV strain (A104) isolated in Styria, Austria, in 1990.

### 3.3. Goat Flock Seroprevalence

The blood of all five goats was taken six weeks after the human TBE case was diagnosed and tested by IFA. One goat showed a positive result with a titer of 80. No reactivity against the other flaviviruses contained in the microchip (West Nile virus, Dengue virus, Yellow fever virus, Japanese encephalitis virus) was found. The result could be confirmed by testing the serum in the neutralization test. A PRNT_90_ titer of 40 was found, which therefore confirmed the IFA result.

## 4. Discussion

Milk-borne TBE is a well-known way of transmission. In the 1950s, the disease was named “biphasic milk fever”, as the alimentary route of transmission was the major epidemiological feature of the disease, with up to hundreds of cases in some outbreaks (e.g., Roznava) [28]. In countries with a highly industrialized milk industry, alimentary TBE cases have been rare. However, in recent years, a number of outbreaks have been recorded in Austria [9], Germany [11], and very recently in France (ProMED-Mail, 27 June 2020). Rarely, a comprehensive study including humans, animals, and ticks has been recorded, and the strain causing TBEV isolated. Here, we present a comprehensive investigation into an alimentary TBEV outbreak in southeastern Germany.

In the current outbreak, only the index patient was severely ill enough to be hospitalized, and the comprehensive retrospective investigation by the local public health office detected 27 exposed people. In 20 of them, who could be contacted and provided information and blood for testing, 13/20 manifested symptomatology compatible with a TBEV infection and a Serological immune response characteristic for an acute TBEV infection. Although a tick-borne infection in some of the infected patients cannot completely be ruled out, it is highly improbable that the infection did not result from the ingestion of contaminated goat milk. As in other alimentary outbreaks, this outbreak also showed a high total manifestation index in the investigated patient group of 65% [14,29].

However, keeping in mind that as 6/20 exposed people were vaccinated against TBE, the true manifestation index is even higher, as only 2/14 non-vaccinated exposed patients did not develop a clinical TBEV infection. The manifestation index of the non-vaccinated sub-group of patients, therefore, is 86%. In an earlier alimentary outbreak in Germany, it could be shown that TBEV can be very unevenly distributed in contaminated goat cheese [11]. This could also be the case for fluid milk when complexes of virus particles form. However, we do not have any information about the amount of goat milk that was ingested by the two non-infected exposed persons. From a goat cheese outbreak in Austria, we know that one person who ate some cheese but then regurgitated it was the only one who did not get infected [9]. Therefore, the amount of ingested virus might be of importance. There is, so far, no information available on the location of this alimentary infection in the body. It can be assumed that the virus has to pass through the highly acid stomach, where it might be possible that lower numbers of ingested TBEV will be inactivated.

Data show that all five completely vaccinated exposed persons were protected against infection. Although only shown in a relatively small number of cases, this is, to the best of our knowledge, the first proof of protection of TBE vaccines against alimentary infection in a larger number of vaccinated and exposed persons, although this was assumed before. Only one vaccinated person has been described to date, who was involved in an alimentary TBEV infection and did not get infected, while three other exposed persons got ill [30]. Moreover, the only vaccinated patient who got ill might not have been protected anymore, as the last vaccination was >15 years ago, and therefore the proposed booster period was exceeded by more than 10 years [31]. Although we know from single cases that, even after >10 years, one vaccine booster might be enough to induce a Serological response and immunity again, this might be different in natural infection, especially in this uncommon way of infection [32].

Only one of the goats was found to be TBEV-antibody-positive; this is in concordance with other data, which show that only a small part of an animal flock might be infected and exhibit antibodies against TBEV, even in highly endemic areas [33,34]. Therefore, we can clearly accuse this goat of being the source of infection. Many studies showed that goats and goat milk are most often involved in transmitting TBEV by the alimentary route, although cattle and sheep might also shed TBEV by their milk [23]. TBEV is actively shed in the milk over the course of four to seven days [35]. The TBE natural focus could be identified by the detection of TBEV in ticks in close proximity to the goat stable, although the stable area and the focus area were separated by a fence. The goat might have been infected by contact with an infected tick directly from the vegetation of focus or by passive transport of infected ticks from the focus to the goats by small rodents or birds. The owner of the goats explained that the goats tried to obtain the tree leaflets outside of their fenced area, and this might have, instead of the fence, brought them into direct contact with infected ticks.

The fact that infected ticks were detectable some weeks after the infection of the goat on two independent sampling activities implies a stable transmission cycle of TBEV in the location and not only a sporadic and random passive introduction of one infected tick by some animals. The apparently low minimal infection rate is characteristic for TBEV, even in ticks directly from the TBEV natural focus [36,37,38]. 

As the goats have been there for several years and, so far, no TBEV infection, either tick-transmitted or by the alimentary route, was reported in the whole region, we speculate that the TBEV was introduced only recently. This idea is supported by the fact that only one goat was seropositive. Therefore, it was interesting to identify the TBEV strain and to analyze the origin of this TBEV.

The phylogenetic analysis of the TBEV E gene sequences directly from the tick pools and the virus strains isolated in A549 cells showed that the two TBEV strains were 100% identical. The sequences were closely related to two sequences found in Emmendingen, a city about 100 km distant from Tübingen and Wutach (Aubachstrasse), a place close to Emmendingen and about 80 km distant to our outbreak area [37]. The next genetically closest of the known strains were isolated in Eastern Bavaria and Austria, several hundred kilometers away. We speculate that the TBEV strain was introduced from a location in the southwestern direction of the focus. Moreover, there might be unidentified TBEV strains in close proximity to the focus area, or the local TBEV strain was introduced by migrating terrestrial animals or birds. Due to the changes in the vegetation structure, the focus did not persist, as no further virus could be detected in the following years.

In conclusion, we report a comprehensive study of an alimentary outbreak of TBE caused by raw goat milk. In accordance with other similar outbreaks reported in recent years, we observed a high infection rate and manifestation index by this route of infection. For the first time, it is proven in a bigger group of exposed people that regular TBE vaccination also protects against the alimentary route of TBEV infection. The TBEV strain seemed to be introduced only recently from an area southwest of the focus. The TBEV probably could establish a stable TBEV transmission cycle for at least some weeks or months. The changes in the local vegetation structure around the goat stable area might have eradicated the focus and caused the disappearance of the TBEV strain from the affected area.

## Data Availability

The obtained sequence was submitted in GenBank.
